# Recent advances in understanding and managing acne

**DOI:** 10.12688/f1000research.25588.1

**Published:** 2020-07-29

**Authors:** Ichiro Kurokawa, Keisuke Nakase

**Affiliations:** 1 Department of Dermatology, Acne Clinical Research Center, Meiwa Hospital, Nishinomiya, Hyogo, 663-8186, Japan; 2 Department of Microbiology, Tokyo University of Pharmacy and Life Sciences, Hachioji, Tokyo, 192-0392, Japan

**Keywords:** Acne, biology, sebaceous gland, hair cycle, gene, immunology, wound healing, post inflammatory hyperpigmentation, vitamin C

## Abstract

Multidisciplinary investigations into the pathogenesis of acne have significantly progressed over the past three years. Studies of the etiology of acne from the perspectives, for example, of sebaceous gland biology, sebum, genetics, keratinization, differentiation, hair cycles, immunology, bacteriology, and wound healing have elucidated its pathogenesis. This has led to the development of new therapies and paved the way for advanced studies that will enable the further evolution of acne treatment.

## Introduction

Acne vulgaris (acne) is an inflammatory disease of the pilosebaceous gland ^1^. It initially forms invisible micro (histopathological) comedones, then often appears in adolescents on the forehead as visible, clinically recognized blackheads or whiteheads (comedones) that develop into inflammatory red papules or pustules. Such lesions can become complicated with either atrophic or hypertrophic scars. Acne can develop into refractory cysts, nodules, and subcutaneous fistulas that are resistant to therapy. Acne most commonly appears on the face, neck, chest, and upper back, where sebaceous follicles predominate. In addition to acne vulgaris, related disorders include follicular occlusive diseases such as acne conglobata, perifolliculitis capitis abscedens et suffodiens (PCAS) (dissecting cellulitis or Hoffman disease), and hidradenitis suppurativa (acne inversa). These are all refractory and therapy-resistant diseases and commonly manifest as hypertrophic scars, cysts, and nodules.

**Figure 1.**
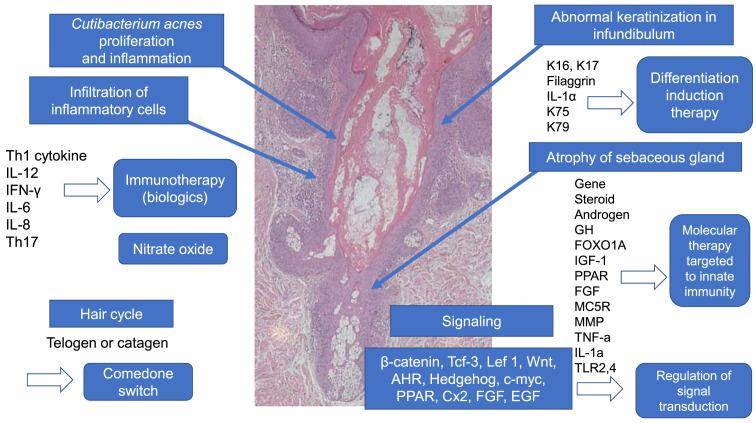
Recent advances in acne pathogenesis. Atrophy of the sebaceous gland is induced by signal transduction due to various factors. In the hair cycle, anagen in normal skin changes to telogen or catagen in the “comedo switch”. Keratin (K) 75 and K79, which are present in the companion layer of normal hair follicles, are reduced in comedo. Immunological aspects show a T helper type 1 (Th1) and Th17 shift in acne. Diversified multiple genes are implicated in acne pathogenesis. According to the above-mentioned etiology, immunity induction therapy, molecular target therapy targeted on innate immunity, and the regulation of signal transduction could be alternative candidates for acne treatment in the future. AHR, aryl hydrocarbon receptor; Cx2, cyclooxygenase-2; EGF, epidermal growth factor; FGF, fibroblast growth factor; FOXO1A, forkhead box transcription factor class O1A; GH, Growth hormone; IFN, interferon; IL, interleukin; Lef, lymphoid enhancer-binding factor; MC5R, melanocortin 5 receptor; MMP, matrix metalloprotease; PPAR, peroxisome proliferation-activated receptor; Tcf, transcription factor; TLR, Toll-like receptor; TNF, tumor necrosis factor; Wnt, Wingless. This image was produced from the author’s clinic for this review.

The following factors are considered to be important to the classical etiology of acne vulgaris ^1^: increased sebum excretion rates, endocrinological factors such as androgens, abnormal keratinization of the follicular infundibulum, bacterial proliferation, and subsequent inflammation. Recent genetic and immunological studies have now elucidated the pathogenesis of acne ( Figure 1).

## Recent findings in the pathogenesis of acne

### Histopathology of acne

Acne is a disease of sebaceous hair follicles that comprises multilobulated large sebaceous acini, tiny vellus hairs, and dilated follicular channels ^2^. Sebaceous follicles are located in the face, chest, and upper back. The sebaceous gland is atrophic in acne lesions ( Figure 1), meaning that sebum has already been discharged into follicular channels because of undifferentiated sebocytes becoming mature and differentiated. Cunliffe proposed cyclical comedo growth and explained the natural resolution of comedones ^3^.

### Recent biology of the sebaceous gland

Stem cells in hair bulges can differentiate into sebocytes and outer root sheaths ^4^. The progenitors of sebaceous glands in the junctional zone between the sebaceous duct and outer root sheath are leucine-rich repeat and immunoglobulin-like domain 1 (LRIG1) ^+^ cells ^5^ that can potentially differentiate into isthmus (epithelial) and sebaceous glands. These cells can also differentiate into sebocytes, sebaceous duct cells, and infundibular keratinocytes depending on the influence of diverse factors. We speculate that undifferentiated sebocytes in acne differentiate into sebaceous duct cells and infundibular keratinocytes instead of mature sebocytes, resulting in the abnormal keratinization of follicular channels. Saurat refers to this phenomenon as a “comedo switch” ^5^. Saurat also stated that not all follicles are involved in acne of the face, neck, and trunk and that only 0.25% of sebaceous glands are involved in visible acne lesions ^5^.

With respect to the molecular network signaling pathway, β-catenin, transcription factor (Tcf) 3, and lymphoid enhancer-binding factor (Lef)-1 are important ^4^. High β-catenin levels stimulate hair follicle formation, whereas low levels stimulate the epidermis and sebaceous gland. Lef-1 cooperates with Indian hedgehog to control the proliferation and differentiation of sebaceous gland progenitors ^4^. Peroxisome proliferation-activated receptor (PPAR), c-Myc, and Cx-2 differentiate towards sebocytes. The repression of β-catenin and activation of c-Myc and the hedgehog signaling cascade promote the differentiation and maturation of sebocytes ^6^.

In addition, Wingless (Wnt) and hedgehog signals are important because the activation of Wnt signaling in LGR5 ^+^ bulge cells promotes hair growth. Loss of Wnt signaling in bulge cells expressing keratin (K) 15 promotes migration and differentiation into sebocytes. Wnt signaling regulates the proliferation and specification of junctional LRIG1 ^+^ cells, resulting in acne pathogenesis ^7^. Androgens might promote sebocyte differentiation and inhibit Wnt signaling ^7^. Aryl hydrocarbon receptors (AHRs) are linked to chloracne ^7^. Exposure to AHR causes comedo formation, sebaceous gland atrophy, and the upregulation of AHR expression in humans and mice ^7^. AHR inhibit sebaceous differentiation by promoting the differentiation of junctional zone stem cells into infundibular keratinocytes ^7^. Fibroblast growth factor (FGF) 2 stimulates proliferation through the pilosebaceous unit ^7^, and epidermal growth factor receptors (EGFRs) can enlarge the hyperproliferation of sebaceous glands and increase sebum production ^7^.

### Sebum

The formation of acne is attributable to a decrease in linoleic acid in classical etiology ^1^. Squalene peroxidated by lipoperoxidase and decreased vitamin E can induce inflammation. Lipoperoxidase can induce pro-inflammatory cytokines and keratinocyte proliferation and activate PPAR ^8^. Monosaturated fatty acids can induce changes in keratinocyte proliferation and differentiation. AHR is metabolized to tetrachlorodibenzodioxin, which induces sebocytes to undergo epithelial-type differentiation. Insulin-like growth factor (IGF) 1 stimulates the formation of unsaturated lipids and neosynthesis of lipids ^9^. The IGF axis is involved in acne pathogenesis ^10^.

Recently, 11β-hydroxysteroid dehydrogenase type I (11β -HSD1) has been observed to promote lipid synthesis. Tumor necrosis factor (TNF)-α promotes lipogenesis in human sebocytes ^11^. Transforming growth factor (TGF)-β maintains sebocytes in an undifferentiated state and decreases lipid accumulation ^12^.

### Genetic findings

Recent genetic findings have clarified that genes encoding the enzymes 21-hydroxylase ( *CYP21A2*), steroid 5α-reductase type I ( *SRD5A1*), and androgen receptor ( *AR*), the somatotropic axis ( *GH1, GHR, IGF1, IGFBP3,* and *IGF1R*), and the forkhead box transcription factor class O1A ( *FOXO1A*), peroxisome proliferator-activated receptor ( *PPARA, PPARB, PPARG, PPARD*), FGF-2 ( *FGF2*), melanocortin receptor ( *MC5R, MC1R*), matrix metalloprotease ( *MMP1, MMP2, MMP3, MMP9, MMP13*), TNF-α ( *TNF*), IL-1α ( *IL1A*), and Toll-like receptors ( *TLR2* and *TLR4*) are implicated in the pathogenesis of acne ^13, 14^.

### Keratinization and hair cycles in acne

Abnormal keratinization is an important factor in acne pathogenesis ^1^. Expression of the hyperproliferative keratins (K6, K16, and K17) is increased in acne lesions ^15^. Significant filaggrin expression in the infundibulum is closely associated with the abnormal keratinization involved in acne ^16^. IL-1α is involved in abnormal keratinization ^17^, and inflammation precedes keratinization ^1^.

Hair cycles in acne have not been studied in detail. van Scott *et al*. stated that the hair cycles in acne are almost always either telogens or catagens ^18^. However, keratin expression in the hair cycle in acne lesions has not been studied. A low microcomedo index in acne is associated with significantly higher K75 expression ^19^. K75 is expressed in the companion layer between the inner (Henle) layer and outer root sheaths in the lower portion of normal hair follicles ^20^. A companion layer is found in anagen but not in either telogen or catagen hair follicles. The prevalence of K75 depends on the hair cycle. Therefore, the hair cycle in acne with microcomedones is directed towards the follicular infundibulum and sebaceous duct instead of the lower portion of hair follicles that comprise companion layers. In addition, K79 is downregulated in comedonal acne lesions ^21^ and is expressed in companion layers in normal anagen hair follicles ^19^. Considering homeostasis in hair cycles in acne, progenitor cells in the junctional zone might differentiate not towards the outer root sheath of hair follicles below the sebaceous duct but towards the infundibulum and sebaceous duct cells in acne.

### Immunological aspects including cytokines

The immunological aspects of acne have become noteworthy. *Malassezia* and *Demodex* are related to the pathogenicity of folliculitis and rosacea, respectively. *Cutibacterium acnes* ( *C. acnes*) and normal flora are involved in acne pathogenesis due to overgrowth in closed follicles. In acne, *C. acnes* in the follicular channel stimulates Langerhans cells in the infundibulum via TLR-2, resulting in the production of IL-12 and IL-8 by activating Th1 cells. *C. acnes* also stimulates follicular keratinocytes in the infundibulum via TLR-2, resulting in the production of IL-6 and IL-8 ^1, 22^ followed by the formation of inflammatory lesions such as red papules and pustules. Jeremy *et al*. proposed that initial inflammation caused by CD4, CD3, and macrophages induces comedones ^23^: inflammation precedes keratinization ^23^. A T helper type 1 (Th1) shift occurs in acne lesions, and Th1-positive cells are more prevalent in acne lesions than in normal skin ^24^. From the aspect of host responses to *C. acnes* in acne pathogenesis, host immunological factors against *C. acnes* produced by PBMCs can be attributed more to bacteriological factors ^25^.

In addition to Th1 cytokines, Th17 is also involved in acne pathogenesis. *C. acnes* is a potent inducer of Th17 and Th1, and significant numbers of cells express IL-17 in acne lesions ^26^. IL-17 is reduced by vitamins A and D. IL-1β and TNF-α are involved in acne inflammation ^11^. Corticotropin-releasing hormone (CRH) can increase IL-6 and IL-8 levels *in vitro* ^12^. Therefore, regulating these cytokines might offer an alternative strategy for treating acne.

### Immunity induction therapy

Acne has been treated with benzoyl peroxide (BPO) formulations, adapalene, antimicrobials, anti-androgen agents, and isotretinoin, which control abnormal follicular keratinization in the infundibulum, have bactericidal and bacteriostatic effects, inhibit inflammation, and decrease sebum excretion, according to the pathomechanism of classical etiological factors. From these immunological aspects in acne, Th1, Th17, and TNF-α are upregulated in acne.

Antimicrobials are useful in immunoregulation because they show not only antibacterial but also anti-inflammatory activities. However, antimicrobial use might increase bacterial resistance to antimicrobials and cause dysbiosis as well as side effects. Novel medicines without these adverse effects have been developed. Vaccination with antibodies against the Christie-Atkins-Munch-Petersen (CAMP) factor, which is associated with *C. acnes* cytotoxicity, decreases the growth of *C. acnes* and the production of murine MIP-2 ^27^. The ability of a vaccine produced by *Staphylococcus capitis* E12 to prevent *C. acnes* overgrowth has been investigated ^28^. A vaccine produced by *S. capitis* is expected for preventing *C. acnes* overgrowth and killing overgrown *C. acnes*. To suppress the inflammation completely, monoclonal antibodies blocking cytokines are needed because killed *C. acnes* induces inflammatory cytokines ^29^.

Th1 and Th17 shift inhibitors and antibodies against IL-17 and TNF-α might offer alternative approaches to treating acne. Notably, a TNF-α antibody is presently used to treat acne conglobata ^30^, hidradenitis suppurativa ^31^, and PCAS ^32^. Antibodies to IL-17, IL-23, and IL-1a will be used to treat acne conglobata, PCAS, and hidradenitis suppurativa, which will also be treated with apremilast ^33^. Nitrate oxide can be an alternative treatment for acne in humans by reducing IL-1β, IL-8, TNF-α, and IL-6 induced by monocytes and IL-8 and IL-6 induced by keratinocytes via innate immunity ^34, 35^.

### Wound healing

The most important complication in acne is scar formation. The rupture and breakdown of inflammatory red papules, pustules, and deep-seated subcutaneous abscesses in the deep dermis can lead to erosion and ulceration, resulting in scar formation. If ulceration is superficial, re-epithelialization proceeds without scarring. However, ulcers located deep below the reticular dermis form scars like deep dermal burns. Atrophic scars have been classified as icepick, boxcar, and rolling scars ^36^, whereas elevated hypertrophic scars like acne conglobata sometimes form. Wound healing in acne should be taken into account when considering scar formation ^37^. Scars form because of persistent inflammation and are associated with the depth of inflammation ^38^. Scar formation is also associated with MMP, IL-6, TGF-β, macrophages, and B cells. Atrophic scars are dependent on B cells and macrophages ^39^. Sebaceous duct cells can differentiate into epidermal keratinocytes and sebocytes in wounds. Thus, sebaceous ducts are bimodal, which is consistent with keratin expression between the infundibulum and sebocytes ^16^.

### Recent non-surgical therapy for postinflammatory hyperpigmentation

Postinflammatory hyperpigmentation (PIH) is an important complication of acne vulgaris that occurs via damage to the basal cell layer. Non-surgical chemical glycolic acid (GA) peels and subsequent iontophoresis using vitamin C, vitamin A, and vitamin E are used to treat PIH, postinflammatory erythema (PIE), and atrophic scars ^37^. GA loosens cellular adhesion, promotes loss from the cornified layer, as well as the regeneration of epidermal and dermal tissues, removes follicular cast in the infundibulum, and de-roofs pustules and red papules ^37^. Vitamin C promotes re-epithelialization while inhibiting melanogenesis and reactive oxygen ^37^. Vitamin C can induce self-renewal of the mesenchymal cell cycle program and fibroblast motility, promote fibroblast migration, confer anti-inflammatory effects, and induce macrophage inflammation ^40^. Both PIH and PIE are treated using the vitamin C derivative, amphipathic vitamin C ^41, 42^. The mechanisms through which vitamin C improves atrophic scars are thought to be *via* self-renewal cell cycle progression, promoted fibroblast migration, matrix deposition and neo-vascularization, anti-inflammatory effects in macrophages, and attenuation mediators in wounds via IL-1β and TNF-α ^40^. In addition, basic FGF (bFGF), an important factor in wound healing, can promote epithelialization and thus improve atrophic scars, resulting in flattening of the epidermis ^43^. bFGF supplies epidermal defects with proliferating keratinocytes.

### Alternative therapy for refractory cysts and nodules in Japan

The pathogenesis of refractory nodulocystic lesions remains unclear. Significant amounts of filaggrin are expressed in cyst formation with retention hyperkeratosis ^44^. Nodulocystic acne and acne conglobata are treated with isotretinoin ^45^, but not in Japan. Kampo (traditional Japanese herbal medicine) such as Saireito can be very effective sometimes for treating nodulocystic acne ^46^ and PCAS ^47^. It works on cystic and alopecia lesions in PCAS, resulting in hair growth ^47^. Hair cycles in PCAS are telogens or catagens as in acne vulgaris. Saireito exerts multiple effects on endogenous corticosteroids, inflammation, reactive oxygen species, coagulation, macrophages, neutrophils, and endothelial cells ^46^. However, the mechanism of Saireito in acne pathogenesis awaits investigation in a basic research study.

### Comprehensive multiplexed therapy in Japan

Isotretinoin and anti-androgen therapy have not been applied in the treatment of acne in Japan. Therefore, severe acne is treated with oral antimicrobials combined with Kampo, topical BPO, and local steroid injections ^48^. Although the mechanism of Kampo is not clear, Saireito can suppress Th1 shift and suppress B cell function, regulating Th1/Th2 balance in mice ^49^. Jyumihaidokuto is available for acne vulgaris in Japan ^50^. It contains Bokusoku, which inhibits rat 5α-reductase activity and testosterone-induced sebum synthesis in hamster sebocytes ^51^. Keigairengyoto is also available for acne vulgaris in Japan ^50^. It has anti-bacterial effects against *C. acnes* ^52^ and inhibits reactive oxygen ^53^. Comprehensive multiplexed therapy is recommended for therapy-resistant refractory acne.

### Future perspectives for acne treatment

This overview describes recent advances in acne pathogenesis. Understanding the multiple diverse, interdependent, and complicated etiological factors and the biology of sebaceous glands is extremely important. Progenitor cells in the junctional zone can differentiate into infundibulum, sebocytes, and outer root sheaths depending on the influence of genes and molecular signals. The altered hair cycle in acne remains of interest from the viewpoint of the biological homeostasis of pilosebaceous units. Immunological studies of acne have generated innovative immunity induction therapies such as antibodies to TNF-α and various cytokines. The most refractory complication of acne is scar formation, which involves the production of pro-inflammatory cytokines such as IL-1α, IL-1β, IL-6, TNF-α, and TGF-β ^54^.

According to the etiopathogenesis of acne, potential treatments for acne in the future are topical anti-androgens, melanocortin receptor antagonists, IGF-1 inhibitors, PPAR modulators, acetylcholine inhibitors, topical retinoic and metabolism-blocking agents, monoclonal antibodies, antimicrobial peptides, antioxidants, phosphodiesterase inhibitors, IL-1β inhibitors, vitamin D analogues, dapsone, systemic antiandrogens, and immunotherapy ^55^.

Immunity induction and wound healing therapies will become key strategies for preventing acne scars. Acne is a disease of the pilosebaceous unit. Based on acne pathogenesis, regulation of sebocyte differentiation is a novel therapeutic procedure ^56, 57^.
